# On the Incubation of Insanity

**Published:** 1845-05

**Authors:** 


*On the Incubation of Insanity.*—Dr. Clutterbuck, at a late meeting of the Medical Society of London, regarded insanity as not a disease *per se*, but merely a symptom of disordered function of the brain. If we admitted—and, he thought, it could not be reasonably doubted—that the brain was the organ through which the mind was manifested, it followed that every disordered condition of the mind was dependent on some disordered condition of the brain, not always, it was true, obvious or appreciable, but still was clear that the brain was not in a sound state of health. Not always to the extent of disorganization, for it was known that insanity often left the patient for a time, and then recurred, from causes not very obvious. The brain was often found diseased in cases of insanity, but we wanted proof that those changes were always the cause of insanity. Authors of experience, however, had asserted that they had always found the brain diseased in cases of insanity; Sutherland and Haslam were of this number; and Mr. Lawrence, out of seventy-two cases, had found the brain diseased in all, a structural change existing in each case. These facts did not prove that the structural disease was the cause of the symptoms, but it showed that in insanity the brain was not sound. That these conditions were not the proximate cause of the insane phenomena, however, was proved, for they existed independent of insanity. We found opacity of the membranes, increased vascularity, bloody points, induration, softening, and serous effusions of the brain, in cases in which insanity did not exist. Changes, however, might exist beyond what we were at present enabled to discover. What, then, caused this state of the brain? He believed that it was always the result of inflammation which had existed at some period or other. He thought this because inflammation was the great disorganizing process; and if disorganized, therefore, the brain must, at one time, have been inflamed. The disorganization was the result, in some way, of inflammation. We might often trace insanity in its early stages to the influence of extreme mental emotion, the effects of alcohol, or of local injuries, the insanity subsiding on the subsidence of these causes, so that we had cause and effect at once before us. He complained that the term incubation was not expressive of the manner in which insanity progressed in its early stages. Confirmed insanity was incurable, as the brain had become permanently affected. The time for treatment was in the early stage; subdue the inflammation then, and you subdue the symptom, and the brain regains its natural condition.—*Lancet.*

